# The correlation between rapid eye movement sleep behavior disorder and the progress of Parkinson’s disease: a systematic review and meta-analysis

**DOI:** 10.3389/fnagi.2024.1418751

**Published:** 2024-07-17

**Authors:** Wentao Zheng, Yang Pan, Kunhua Li, Keyu Tao, Qiuyu Wang, Yungui Yang

**Affiliations:** Internal Medicine, Qujing Third People’s Hospital, Qujing, Yunnan, China

**Keywords:** Parkinson’s disease, rapid eye movement sleep behavior disorder, motor symptom, non-motor symptom, meta-analysis

## Abstract

**Background:**

This meta-analysis was conducted to evaluate potential differences in symptoms between PD patients with or without RBD.

**Methods:**

A systematic search was conducted in PubMed, Cochrane, Embase, and Web of Science databases (as of August 16, 2023), to identify relevant studies on PD and RBD. Statistical analysis was performed using Stata 15.0. Continuous variables were analyzed using the standardized mean difference (SMD) and 95% confidence interval (95% CI), while count data were assessed using the odds ratio (OR) and 95% CI as statistical effect sizes. Heterogeneity among all included studies was tested; for studies with low heterogeneity (I^2^ < 50%), a fixed-effects model was used to calculate statistical results. For studies with relatively high heterogeneity (I^2^ > 50%), a random-effects model was applied, followed by sensitivity and subgroup analyses to identify sources of heterogeneity.

**Results:**

A total of 5,672 subjects were involved in this study. Compared to the NRBD group, the UPDRS-III score in the RBD group was significantly higher (SMD = 0.20, 95% CI: [0.11, 0.29], *P* < 0.001), and the Hoehn-Yahr score in the RBD group was also significantly higher (SMD = 0.29, 95% CI: [0.03, 0.55], *P* < 0.001). Patients with PD in the RBD group had more severe cognitive impairments than those in the NRBD group (SMD = −0.30, 95% CI: [−0.48, −0.11], *P* < 0.001). The incidence of hallucination in PD patients in the RBD group was 3.0 times that of the NRBD group (OR = 3.0, 95% CI: [2.15, 4.20], *P* = 0.110). PD patients in the RBD group also experienced more severe anxiety symptoms (SMD = 0.13, 95% CI: [−0.26, 0.51], *P* < 0.001), had higher scores in depression scales (SMD = 0.22, 95% CI: [0.02, 0.43], *P* < 0.001), and higher scores in sleep disorder scales than those in NRBD group (SMD = 0.10, 95% CI: [−0.11, 0.31], *P* < 0.001).

**Conclusion:**

Results show PD patients with co-occurring RBD have more severe motor and non-motor symptoms likely due to overlapping affected regions in RBD and PD-related pathology, plus broader neurodegeneration seen in PD patients with RBD.

**Systematic review registration:**

https://www.crd.york.ac.uk/PROSPERO/#searchadvanced, identifier CRD42023476331.

## 1 Introduction

Parkinson’s disease (PD) is a common neurodegenerative disorder that predominantly affects the elderly, characterized by both motor and non-motor symptoms (Cabreira and Massano, 2019). The motor symptoms include resting tremors, rigidity, and bradykinesia, while the non-motor symptoms mainly manifest as cognitive impairments, sleep disorders, and anxiety and depression disorders (Hayes, 2019). The global prevalence of PD is reported to be 1/1000, and the incidence increases with age (Tysnes and Storstein, 2017). The pathogenesis of PD primarily involves the degeneration of the nigrostriatal pathway, leading to a reduction in the dopamine level and a relative excess of cholinergic activity (Raza et al., 2019).

Sleep disorder is among the most common non-motor symptoms of PD, with rapid eye movement sleep behavior disorder (RBD) being a prevalent abnormal sleep behavior. According to the 3rd International Classification of Sleep Disorders (ICSD-3), diagnosis of RBD is based on the presence of repeated episodes of vocalization or complex motor behaviors during REM sleep in polysomnography (PSG) and on polysomnographic recording of REM sleep without atonia (RSWA). RBD is commonly observed in neurodegenerative diseases such as multiple system atrophy (MSA), Lewy body dementia (LBD), and PD (Pilotto et al., 2019). Previous studies have identified that RBD may appear as early as the early stages of PD, which often presents approximately 10 years before the motor symptoms of PD patients and could be a precursor symptom of PD. According to relevant statistics, the prevalence of RBD in PD patients ranges from 25 to 60% (Arnulf, 2012). Recent evidence suggests that the presence of RBD is associated with differences in the clinical symptom spectrum, natural history, and prognosis of PD. These differences are likely indicative of underlying differences in the pathophysiology among PD patients with and without RBD.

Currently, existing studies have analyzed and compared the clinical characteristics of PD patients with and without RBD. The results have shown that PD patients with RBD may have an increased risk of non-motor symptoms such as anxiety, depression, sleep disorder, hallucination, and cognitive impairments. Furthermore, patients with RBD often exhibit more severe motor symptoms, including abnormalities in posture and gait, language expression difficulty, and increased resting tremor duration. However, these articles are somewhat outdated. In recent years, new studies on PD patients and RBD have emerged, yielding inconsistent results that have sparked our interest. Previous meta-analyses on PD and RBD were constrained by the number of original articles and varying research focuses, lacking comprehensive and meticulous analysis of RBD’s impact on both motor and non-motor symptoms in PD patients, including sleep conditions and anxiety levels. Moreover, these studies lacked thorough subgroup analyses, potentially introducing bias into their findings. Therefore, we conducted a systematic search and collected literature to integrate all relevant studies using meta-analysis. Through the expansion of sample size, we systematically analyzed the progression of PD with and without RBD and their clinical outcomes, so as to provide effective evidence-based medical insights to clarify the precise relationship between RBD and PD.

## 2 Method

This study strictly followed the PRISMA guidelines (Page et al., 2021). Besides, Its protocol was registered in the PROSPERO (registration ID: CRD42023476331).

### 2.1 Search strategy

Databases including PubMed, the Cochrane Library, Embase, and Web of Science were searched and records were limited to the English literature published before August 2023. The search strategy was designed by two researchers (Wentao Zheng and Yungui Yang) independently using the combination of Medical Subject Heading (MeSH) terms and their free words (i.e., “REM sleep behavior disorder” OR “rapid eye movement behavi*” OR “rapid eye movement sleep behavi*” OR “rapid eye movement sleep behavior disorder” OR“REM behavi*” OR “REM sleep behavi*” and “Parkinson disease” OR “Parkinson’s disease” OR “Primary Parkinsonism”). The full search history is presented in Supplementary Table 1. Due to the small number of the recently updated literature, we did not update the data analysis further. Any disagreement arising during the study shall be decided by arbitration by a third researcher (Yang Pan).

### 2.2 Inclusion and exclusion criteria

The included studies must meet the following criteria: (1) The study population should be patients with primary PD, who must be diagnosed according to the U.K. Parkinson’s Disease Society Brain Bank (PDSBB) criteria (Gelb et al., 1999) and other published criteria (Postuma and Berg, 2017; Cabreira and Massano, 2019); (2) Studies should focus on the clinical features of RBD-related PD, such as the study by Lee (Lee et al., 2010), which compared differences in the Hoehn and Yahr stage and Unified Parkinson’s Disease Rating Scale (UPDRS) scores between PD patients with and without RBD. The study also assessed common non-motor symptoms and cognitive functions in PD patients to explore the correlation between RBD and the clinical severity of PD; (3) The diagnosis of RBD must meet the diagnostic criteria of the International Classification of Sleep Disorders. Patients diagnosed with polysomnography (PSG) are considered to have confirmed RBD (cRBD), while those diagnosed based on interviews or questionnaires are considered to have confirmed probable RBD (pRBD) (St Louis and Boeve, 2017); (4) Studies must be published in English to be included.

Exclusion criteria for the study are: (1) Original research articles not covering the clinical features of RBD-related PD; (2) Meta-analyses/reviews, conference abstracts, guidelines, letters, responses, editorial materials, case reports, and animal experiments; (3) Duplicates, articles only featuring RBD characteristics, and literature on the pathogenesis of PD with RBD; (4) Articles with missing data or inaccessible data; (5) Studies with non-standard PD and RBD diagnostic criteria or poor research quality; (6) Studies reported in languages other than English.

### 2.3 Study selection

Based on the previously established eligible criteria, two researchers (Wentao Zheng and Yungui Yang) independently conducted the study selection. First, all potentially relevant articles retrieved from databases were imported into EndNote v9.0 for deduplication. Next, articles that did not match the research content were excluded based on their titles and abstracts. Finally, the full texts were further screened, with any disagreements resolved through discussion or consultation with a third researcher.

### 2.4 Data extraction

Two researchers (Wentao Zheng and Yungui Yang) independently collected relevant literature and extracted the required research information using a pre-designed datasheet. The extracted information included the first author, year of publication, country of publication, study type, diagnosis criteria, sample size, sex, age, and outcome indicators. Outcome indicators for motor symptoms included Hoehn and Yahr score and UPDRS-III score and for non-motor symptoms included mental health score (anxiety, depression), cognitive function score, sleep score, and the incidence of visual hallucinations. Any disputes during the extraction process were resolved by discussion with a third researcher.

### 2.5 Quality assessment

To assess the bias and quality of the included studies, the two researchers used the Newcastle-Ottawa Scale (NOS) (Wells et al., 2014) for quality evaluation, employing a semi-quantitative star rating system with a maximum score of 10 stars. Studies were categorized into very high risk of bias (0–3 stars), high risk of bias (4–6 stars), and low risk of bias (7–9 stars), with studies scoring ≥5 stars considered of high quality and eligible for inclusion in this meta-analysis. Any disagreements were resolved through discussion or negotiation with a third researcher.

### 2.6 Statistical analysis

All data were statistically analyzed by the professional meta-analysis software Stata 15.0. Continuous variables were analyzed using the standardized mean difference (SMD) and 95% confidence interval (95% CI), while count data were assessed using the odds ratio (OR) and 95% CI as statistical effect sizes. Heterogeneity among all included studies was tested; for studies with low heterogeneity (I^2^ < 50%), a fixed-effects model was used to calculate statistical results; for studies with significant heterogeneity (I^2^ > 50%), a random-effects model was applied, followed by sensitivity and subgroup analyses to identify sources of heterogeneity. To explore potential publication bias, if the number of included clinical study articles met the analysis criteria (≥8 studies included), funnel plot analysis would be conducted along with Begg’s test for statistical testing (Egger et al., 1997), to determine the presence of publication bias. If the funnel plot appeared symmetrical and the Begg’s test yielded a *P* value > 0.05, it would be considered that there was no significant publication bias.

## 3 Results

### 3.1 Retrieval results and study characteristics

**FIGURE 1:**
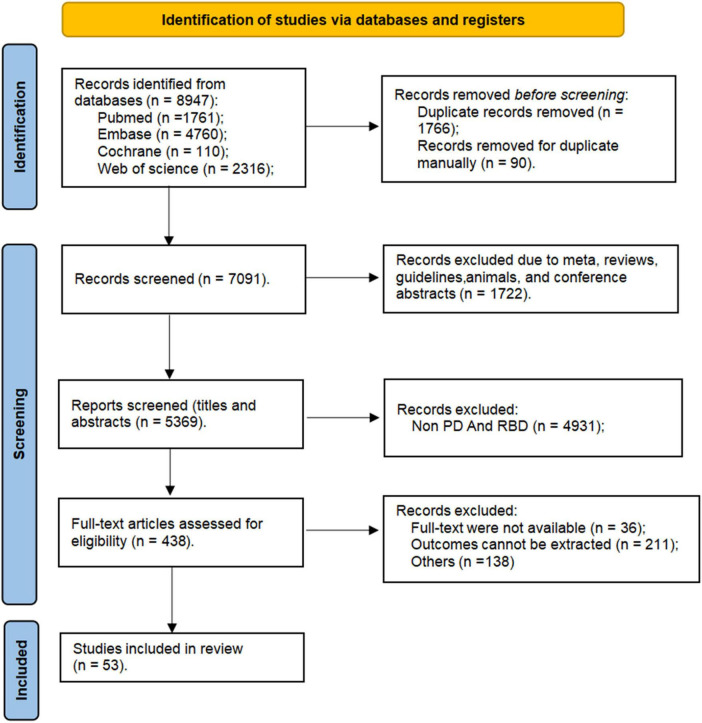
Study selection flow chart.

In this study, a search of relevant databases initially identified a total of 8,947 articles (Figure 1) from PubMed (*n* = 1761), Embase (*n* = 4760), Cochrane (*n* = 110), and Web of Science (*n* = 2316). Initially, 1,856 duplicates were removed automatically and manually using EndNote v9.0, followed by the exclusion of 1,722 articles of types such as meta-analyses, reviews, guidelines, animals, and conference abstracts. Through reviewing titles and abstracts, 4931 articles were excluded as they did not simultaneously include features related to both PD and RBD. Articles were further eliminated for reasons such as full-text were not available (*n* = 36), outcomes cannot be extracted (*n* = 211), and non-compliance with inclusion criteria (*n* = 138), leaving a total of 53 articles included in our study.

In the 53 studies included in this study, the RBD group comprised a total of 3,084 patients, with 1,265 patients diagnosed with cRBD (average age: 57.3–76.5 years; 777 males and 488 females) and 1,819 diagnosed with pRBD (average age: 60.38–73.1 years; 1,124 males and 695 females). The NRBD group included a total of 4,362 patients (average age: 57.1–74.5 years; 2,422 males and 1,940 females). The diagnosis of PD in the 53 studies was primarily based on the PDSBB criteria (Hughes et al., 1992). The diagnostic criteria for cRBD mainly followed the International Classification of Sleep Disorders and PSG (St Louis and Boeve, 2017), while pRBD diagnosis was chiefly based on the REM Sleep Behavior Disorder Screening Questionnaire (Stiasny-Kolster et al., 2007). Specific diagnostic criteria are listed in Table 1. Geographically, 14 studies originated from Europe (Sinforiani et al., 2006; Benninger et al., 2010; Lavault et al., 2010; Bugalho et al., 2011; Bugalho and Viana-Baptista, 2013; Ferri et al., 2014; Rolinski et al., 2014; Mariotti et al., 2015; Arnaldi et al., 2016; Leclair-Visonneau et al., 2017; Bargiotas et al., 2019; Figorilli et al., 2020; Assogna et al., 2021; Oltra et al., 2021), 23 from Asia (Ozekmekçi et al., 2005; Meral et al., 2007; Yoritaka et al., 2009; Lee et al., 2010; Vibha et al., 2011; Aygün et al., 2012; Nihei et al., 2012; Nomura et al., 2013, 2016, 2017; 2020; Suzuki et al., 2013; Gong et al., 2014; Kim et al., 2014; Hu et al., 2015; Kang et al., 2016; Kamble et al., 2019; Liu et al., 2019; Yan et al., 2019; Cao et al., 2020; Ashraf-Ganjouei et al., 2021; Zhu et al., 2021; Fujita et al., 2022), 15 from North America (Gagnon et al., 2004, 2009; Postuma et al., 2008, 2009, 2011; 2012; Gaudreault et al., 2013; Neikrug et al., 2014; Chahine et al., 2016; Pagano et al., 2018; Sobreira-Neto et al., 2018; Duarte Folle et al., 2019; Trout et al., 2019; Mahmood et al., 2020; Yoon and Monchi, 2021), and 1 from Oceania (Ford et al., 2013). Among the included studies, 49 reported outcome measures related to motor symptoms [e.g., UPDRS-III: Paulo Bugalho (Bugalho and Viana-Baptista, 2013); Hoehn and Yahr stage: Raffaele Ferri (Ferri et al., 2014)], while 39 reported outcome measures related to non-motor symptoms such as anxiety (Paolo Mariotti) (Mariotti et al., 2015), depression (L.M. Chahine) (Chahine et al., 2016), sleep disorder (Gennaro Pagano) (Pagano et al., 2018), hallucinations (L.M. Chahine) (Chahine et al., 2016), and Mini-Mental State Examination (MMSE) (Jun Zhu) (Zhu et al., 2021).

**TABLE 1:** Characteristics of studies included in the meta-analyses.

References	Country	Sample size (n)	RBD assess-ment	Diagno-sis criteria	RBD		NRBD		Outcomes	NOS score
					**Gender** **(M/F)**	**Age** **(mean ± SD)**	**Gender** **(M/F)**	**Age** **(mean ± SD)**		
Bugalho and Viana-Baptista, 2013	Portugal	75	ICSD-R	PD Criteria8	–	–	–	–	UPDRS-III	7
Aygün et al., 2012	Samsun	141	ICDS-R	PDSBB	60/30	67.14 ± 9.9	31/20	65.00 ± 12	UPDRS-III	7
Meral et al., 2007	Turkey	38	ICSD	PDSBB	13/8	67.6 ± 8.9	10/7	68.9 ± 7.3	UPDRS-III, hallucination	7
Mariotti et al., 2015	Italy	85	PSG	PDSBB	33/16	68.3 ± 7.05	18/18	69.0 ± 9.63	UPDRS-III, anxiety, depression, sleep disorder	9
Postuma et al., 2012	Canada	42	PSG	movement disorders specialist	23/4	70.5 ± 7.4	11/4	67.5 ± 10.6	UPDRS-III, MMSE, hallucination	9
Postuma et al., 2008	Canada	36	ICSD-II	PDSBB	17/4	68.0 ± 8.6	8/7	65.5 ± 9.3	UPDRS-III	9
Sinforiani et al., 2006	Italy	66	ICSD	PDSBB	19/12	63.0 ± 8.17	23/12	66.03 ± 9.19	UPDRS-III, MMSE	10
Leclair-Visonneau et al., 2017	France	45	PSG	PDSBB	22/8	62.1 ± 6.2	8/7	57.1 ± 9.0	UPDRS-III	9
Benninger et al., 2010	Switzerland	26	PSG	PDSBB	11/2	63.1 ± 8.0	10/3	62.2 ± 7.7	UPDRS-III, H-Y, sleep disorder, hallucination	8
Sobreira-Neto et al., 2018	Brazil	66	PSG	–	30/25	60.4 ± 10.6	6/5	58.4 ± 16.1	UPDRS-III, H-Y, MMSE, sleep disorder	9
Vibha et al., 2011	India	134	ICSD-R	PDSBB	19/7	61.2 ± 8.56	71/37	57.57 ± 11.85	UPDRS-III, MMSE, sleep disorder, hallucination	6
Neikrug et al., 2014	USA	66	PSG	PDSBB	25/11	67.25 ± 7.3	18/8	68.38 ± 10.0	UPDRS-III	6
Gagnon et al., 2009	Canada	/	ICSD-II	PDSBB	/	66.4 ± 8.47	/	65.69 ± 8.52	UPDRS-III, H-Y, depression, sleep disorder	9
Postuma et al., 2009	Canada	55	PSG	PDSBB	28/6	69.4 ± 10.76	13/8	68.70 ± 9.00	UPDRS-III, MMSE	10
Zhu et al., 2021	China	172	PSG	PDSBB	57/36	66.9 ± 7.16	44/35	64.99 ± 6.39	UPDRS-III, H-Y, MMSE, anxiety, depression, sleep disorder	6
Kamble et al., 2019	India	/	PSG	PDSBB	/	57.3 ± 6.6	/	60.4 ± 8.2	UPDRS-III, MMSE	9
Assogna et al., 2021	Italy	76	PSG	PDSBB	31/7	67.16 ± 7.38	31/7	67.26 ± 7.18	UPDRS-III, H-Y, MMSE	9
Gong et al., 2014	China	112	PSG	PDSBB	40/23	66.8 ± 7.80	30/19	65.3 ± 11.03	UPDRS-III, H-Y, MMSE, sleep disorder, hallucination	9
Bugalho et al., 2011	Portugal	65	ICSD	PD Criteria (Gelb et al., 1999)	15/26	72.3 ± 7.04	17/17	72.8 ± 7.44	UPDRS-III, H-Y	6
Postuma et al., 2011	Canada	53	ICSD-II	Not reported	27/5	67.9 ± 9.0	11/10	67.3 ± 8.0	UPDRS-III	9
Bargiotas et al., 2019	Switzerland	/	PSG	Not reported	/	62.6 ± 7.7	/	63.0 ± 9.4	UPDRS-III, H-Y, MMSE, depression, sleep disorder	6
Gaudreault et al., 2013	Canada	200	PSG	PDSBB	69/31	64.7 ± 8.0	60/40	63.1 ± 6.0	UPDRS-III, H-Y, MMSE	9
Ferri et al., 2014	UK	21	PSG	PDSBB	5/5	67.8 ± 8.28	7/4	66.5 ± 6.38	H-Y, MMSE	7
Kim et al., 2014	Korea	448	ICSD-R	PDSBB	122/144	64.6 ± 8.79	90/92	62.18 ± 9.97	MMSE	6
Nomura et al., 2020	Japan	51	PSG	PDSBB	17/15	71.4 ± 7.2	6/13	66.9 ± 9.0	H-Y, MMSE	9
Nomura et al., 2017	Japan	136	PSG	PDSBB	26/21	73.1 ± 7.3	35/54	71.1 ± 7.8	MMSE, hallucination	9
Gagnon et al., 2004	Canada	15	PSG	Not reported	6/1	68.4 ± 7.5	3/5	61.0 ± 7.3	H-Y, MMSE, depression	6
Figorilli et al., 2020	France	44	PSG	PDSBB	17/5	64.0 ± 6.9	17/5	67.1 ± 6.8	Sleep disorder	9
Yoritaka et al., 2009	Japan	150	ICSD-R	Not reported	45/36	70.6 ± 8.1	25/44	66.0 ± 11.1	H-Y, hallucination	7
Ashraf-Ganjouei et al., 2021	Iran	420	RBDSQ	Not reported	110/48	61.92 ± 9.65	165/97	61.49 ± 9.77	UPDRS-III	8
Ford et al., 2013	Australian	126	MSQ	PDSBB	36/12	66.4 ± 9.9	48/30	65.8 ± 10.9	UPDRS-III, MMSE, depression, sleep disorder	7
Nihei et al., 2012	Japan	469	RBDSQ-J	PDSBB	67/79	71.7 ± 8.2	152/171	70.6 ± 8.8	UPDRS-III, H-Y	7
Suzuki et al., 2013	Japan	77	RBDSQ-J	PDSBB	9/9	71.4 ± 5.0	31/28	68.3 ± 9.8	UPDRS-III, H-Y, depression, sleep disorder	6
Yoon and Monchi, 2021	Canada	81	RBDSQ	Not reported	2/9	64.9 ± 7.9	26/44	62.3 ± 7.2	UPDRS-III, anxiety, depression	7
Chahine et al., 2016	USA	423	RBDSQ	Not reported	79/29	61.90 ± 9.86	198/117	61.65 ± 9.70	UPDRS-III, depression, hallucination	6
Hu et al., 2015	China	210	RBDSQ	PDSBB	38/29	60.91 ± 11.69	77/66	59.59 ± 10.63	UPDRS-III	9
Oltra et al., 2021	Spain	59	5-item test	PDSBB	23/4	68.8 ± 9.2	21/11	64.5 ± 9.9	UPDRS-III, MMSE	7
Duarte Folle et al., 2019	USA	778	RBDSQ	Not reported	127/38	70 ± 9.6	368/245	70.6 ± 10.4	UPDRS-III	6
Yan et al., 2019	China	89	The RBD-HK questionnaire	MDS	21/25	66.18 ± 3.17	19/24	64.71 ± 4.34	UPDRS-III, H-Y, MMSE	6
Fujita et al., 2022	Japan	126	ICSD-R + RBDSQ-J	MDS	17/14	71.3 ± 7.1	40/55	68.4 ± 9.5	UPDRS-III, H-Y, MMSE, depression, sleep disorder	9
Nomura et al., 2016	Japan	70	RBDSQ-J	PDSBB (Daniel and Lees, 1993)	15/12	68.8 ± 8.6	16/27	69.4 ± 9.2	H-Y, MMSE	7
Lee et al., 2010	Korea	447	ICSD-R	PDSBB	89/75	65.1 ± 8.4	128/155	63.1 ± 9.6	H-Y, MMSE	6
Trout et al., 2019	USA	50	RBDSQ	UPDRS (Gelb et al., 1999)	16/3	70.1 ± 7.4	22/9	65.7 ± 6.6	H-Y, anxiety, sleep disorder	7
Liu et al., 2019	China	158	RBDSQ	PDSBB	61/66	60.38 ± 10.84	14/17	58.32 ± 10.36	UPDRS-III, anxiety, depression, sleep disorder	10
Rolinski et al., 2014	UK	475	RBDSQ	PDSBB	128/96	67.5 ± 9.4	165/86	67.9 ± 9.5	UPDRS-III, H-Y, MMSE, depression	9
Pagano et al., 2018	Canada	272	RBDSQ	Not reported	69/37	61.8 ± 9.68	63/103	61.47 ± 9.78	UPDRS-III, depression, sleep disorder	9
Mahmood et al., 2020	USA	123	RBDSQ	PDSBB	43/15	68.40 ± 8.31	43/22	66.88 ± 8.42	UPDRS-III, H-Y, anxiety, depression	9
Arnaldi et al., 2016	Italy	38	MSQ	PD Criteria8	9/5	72.8 ± 6.2	15/9	70.6 ± 7.1	UPDRS-III, MMSE	7
Lavault et al., 2010	France	61	RBDSQ	PDSBB	26/13	66.6 ± 7.9	13/9	60.3 ± 11.1	UPDRS-III, MMSE, depression, sleep disorder, hallucination	9
Nomura et al., 2013	Japan	59	Not reported	Not reported	14/13	76.5 ± 5.9	14/18	74.5 ± 8.1	H-Y, MMSE, hallucination	6
Kang et al., 2016	Korea	96	RBDSQ	PDSBB	20/23	71.80 ± 2.23	21/32	72.26 ± 7.73	MMSE	7
Cao et al., 2020	China	320	RBDSQ	Not reported	78/33	61.9 ± 9.7	133/76	61.8 ± 9.5	UPDRS-III, MMSE, anxiety, depression	9
Ozekmekçi et al., 2005	Turkey	70	Not reported	Not reported	27/8	67.2 ± 8.6	27/8	67.61 ± 9.91	UPDRS-III	6

RBD, rapid eye movement sleep behavior disorder; PRBD, diagnosis based on interview/questionnaires, no PSG reconfirmation; PDSBB, U.K. Parkinson’s Disease Society Brain Bank criteria; H-Y, Hoehn and Yahr score; ICSD, the International Classification of Sleep Disorders criteria; ICSD-R, International Classification of Sleep Disorders Revised; MMSE, Mini Mental State Examination; RBDSQ, the RBD screening questionnaire; RBDSQ-J, REM Sleep Behavior Disorder Screening Questionnaire-Japanese; RBDQ-HK, REM Sleep Behavior Disorder Questionnaire-Hong Kong; MSQ, Mayo Sleep Questionnaire; PSG, Polysomnography;5-item test:5-item test Inns-bruck REM Sleep Behavior Disorder Inventory; MSQ, Mayo Sleep Questionnaire.

### 3.2 Quality assessment

Two researchers conducted a quality assessment of the included articles using the NOS (Wells et al., 2000), which evaluates three aspects: (1) the selection methods of cases and controls, (2) the comparability between case and control groups, (3) the methods of assessing exposure. The scores from these three evaluations are summed, with a higher total score indicating better quality, and the maximum possible score being 10. In this study, the NOS scores ranged from a high of 10 (Sinforiani et al., 2006; Postuma et al., 2009; Liu et al., 2019) to a low of 6 (Gagnon et al., 2004; Ozekmekçi et al., 2005; Lee et al., 2010; Bugalho et al., 2011; Vibha et al., 2011; Nomura et al., 2013; Suzuki et al., 2013; Kim et al., 2014; Neikrug et al., 2014; Chahine et al., 2016; Bargiotas et al., 2019; Duarte Folle et al., 2019; Yan et al., 2019; Zhu et al., 2021), indicating generally high quality of the included studies.

### 3.3 Differences in motor symptoms in PD patients with and without RBD

#### 3.3.1 Analysis of UPDRS-III scores

**FIGURE 2:**
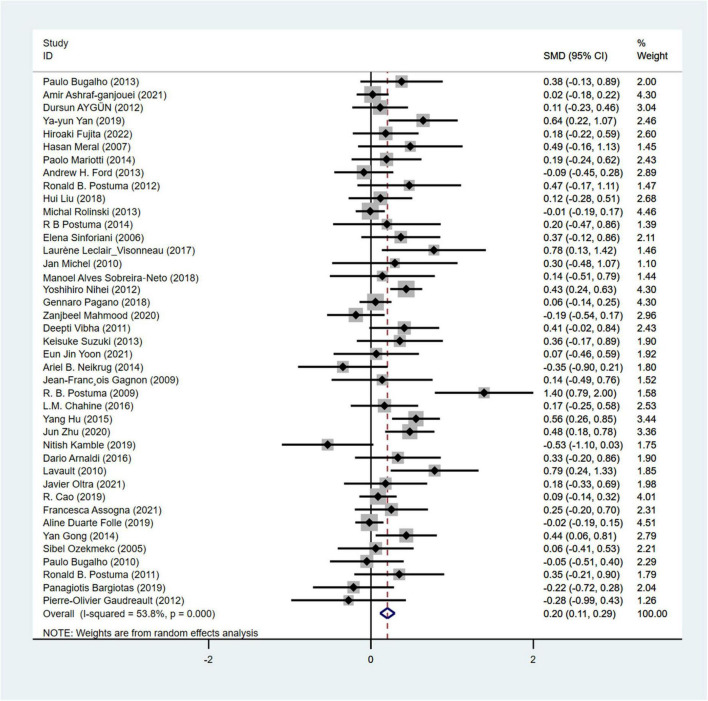
Forest plot based on difference in the UPDRS-III score among Parkinson’s disease patients with or without rapid eye movement sleep behavior disorder (RBD). SMD, Standardized Mean Difference; CI, confidence intervals.

UPDRS-III was used in a total of 41 articles (Sinforiani et al., 2006; Meral et al., 2007; Postuma et al., 2008, 2009, 2012; Gagnon et al., 2009; Benninger et al., 2010; Vibha et al., 2011; Aygün et al., 2012; Bugalho and Viana-Baptista, 2013; Neikrug et al., 2014; Mariotti et al., 2015; Leclair-Visonneau et al., 2017; Sobreira-Neto et al., 2018; Snyder et al., 2021; Zhu et al., 2021) involving 5,976 PD patients to assess the severity of motor symptoms (cRBD group: 2,392 patients; NRBD group: 3,584 patients). Due to high heterogeneity among the included studies (I^2^ = 53.8%), a random-effects model was used for analysis. The results showed that the UPDRS-III scores were significantly higher in the PD with RBD group compared to the PD without RBD group (SMD = 0.20, 95% CI: [0.11, 0.29], *P* < 0.001), (Figure 2) indicating more severe motor symptoms in PD patients with RBD. Subgroup analyses were conducted based on RBD diagnosis, geographic regions, and RBD types. According to the results, by RBD types, the heterogeneity I^2^ was 47.7% for the cRBD group and 53.8% for the pRBD group; by geographic regions, I^2^ was 35.7% for Europeans, 55.4% for Asians, and 56.1% for Americans; by RBD diagnosis, I^2^ was 0.0% for ICSD, while I^2^> 50% for other diagnostic tools like PSG and RBDSQ, indicating that RBD diagnosis, geographic regions, and RBD types might be the main sources of heterogeneity in UPDRS-III score analysis. Additionally, subgroup analysis results (Supplementary Table 2) showed that UPDRS-III scores were higher in PD patients with cRBD or pRBD compared to the control group of PD patients (PD with RBD, SMD = 0.24; PD with pRBD, SMD = 0.20). The funnel plot (Supplementary Figure 1) and the Begg’s test (*P* = 0.181) indicated no significant publication bias.

#### 3.3.2 Analysis of Hoehn and Yahr stage

**FIGURE 3:**
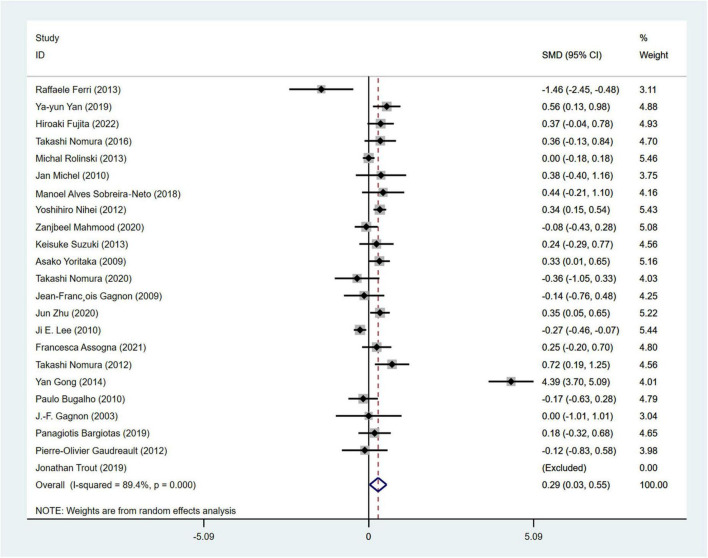
Forest plot based on the difference in Hoehn and Yahr stage among Parkinson’s disease patients with or without rapid eye movement sleep behavior disorder (RBD). SMD, Standardized Mean Difference; CI, confidence intervals.

Hoehn and Yahr stage was used in a total of 23 articles involving 3,557 PD patients (RBD group: 1,630 patients, NRBD group: 1,927 patients) to assess the severity of PD. The heterogeneity test showed relatively high variability among the studies (I^2^ = 89.4%), thus a random-effects model was employed for the analysis. The results indicated that the Hoehn-Yahr score was higher in the PD with RBD group compared to the PD without RBD group (SMD = 0.29, 95% CI: [0.03, 0.55], *P* < 0.001) (Figure 3). Sensitivity analysis demonstrated the stability of the studies included in this meta-analysis with no identifiable source of heterogeneity. Subgroup analysis by geographic regions (Supplementary Table 2) showed that the geographic origin of the population might be a source of heterogeneity in the analysis of the Hoehn and Yahr stage (Americans: I^2^ = 0.0%, Europeans: I^2^ = 56.7%, Asians: I^2^ = 94.3%). The funnel plot (Supplementary Figure 1) and Begg’s test (*P* = 0.612) indicated no publication bias.

### 3.4 Differences in non-motor symptoms in PD patients with and without RBD

#### 3.4.1 Analysis of cognitive function performance

**FIGURE 4:**
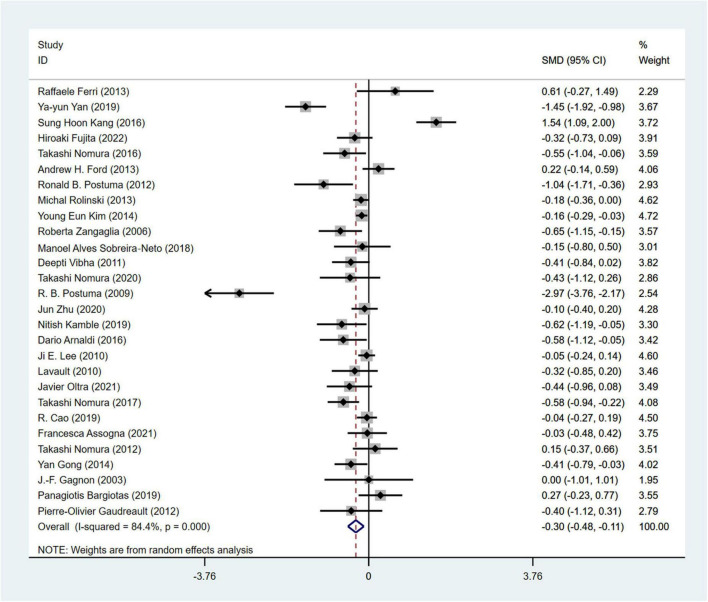
Forest plot based on the difference in MMSE score among Parkinson’s disease patients with or without rapid eye movement sleep behavior disorder (RBD). SMD, Standardized Mean Difference; CI, confidence intervals.

MMSE (Snyder et al., 2021) was used in a total of 28 studies (Sinforiani et al., 2006; Lee et al., 2010; Vibha et al., 2011; Ferri et al., 2014; Gong et al., 2014; Kim et al., 2014; Bargiotas et al., 2019; Kamble et al., 2019; Nomura et al., 2020; Assogna et al., 2021; Zhu et al., 2021) involving 2,946 PD patients (RBD group: 1,293 patients, NRBD group: 1,653 patients) to evaluate cognitive impairments. The heterogeneity among the studies was high (I^2^ = 84.4%), and a random-effects model was employed for the analysis. PD patients in the RBD group exhibited more severe cognitive impairments than those in the NRBD group (SMD = −0.30, 95% CI: [−0.48, −0.11], *P* < 0.001) (Figure 4). Subgroup analysis (Supplementary Table 3) suggested that heterogeneity might stem from geographic regions, with I^2^ being 47.7% for Europeans, 87.1% for Asians, and 89.0% for North Americans. The funnel plot (Supplementary Figure 2) and Begg’s test (*P* = 0.228) indicated no publication bias, and sensitivity analysis suggested good stability across the studies.

#### 3.4.2 Analysis of hallucination incidence

**FIGURE 5:**
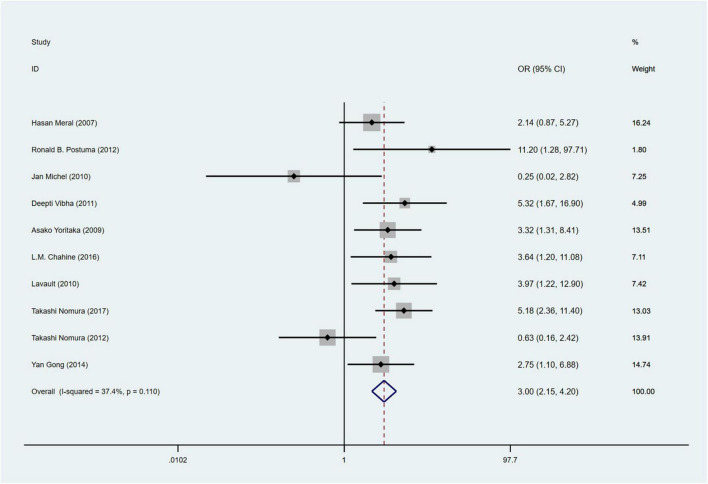
Forest plot based on the odds ratio (OR) for incidence of hallucinations in Parkinson’s disease patients with or without rapid eye movement sleep behavior disorder (RBD). CI, confidence intervals.

A total of 10 studies (Meral et al., 2007; Yoritaka et al., 2009; Benninger et al., 2010; Lavault et al., 2010; Vibha et al., 2011; Postuma et al., 2012; Nomura et al., 2013, 2017; Gong et al., 2014; Chahine et al., 2016) involving 1,168 PD patients were included in the meta-analysis for the incidence of hallucinations (RBD group: 439 patients, NRBD group: 729 patients). The heterogeneity among the studies was relatively low (I^2^ = 37.4%), so a fixed-effects model was employed for integration. The results indicated that the incidence of hallucinations in PD patients in the RBD group was 3.0 times greater than that in the NRBD group (95% CI: [2.15, 4.20], *P* = 0.110) (Figure 5). The funnel plot (Supplementary Figure 2) yielded symmetrical patterns among the studies, and Begg’s test (*P* = 0.858) suggested no publication bias among them.

#### 3.4.3 Analysis of anxiety

**FIGURE 6:**
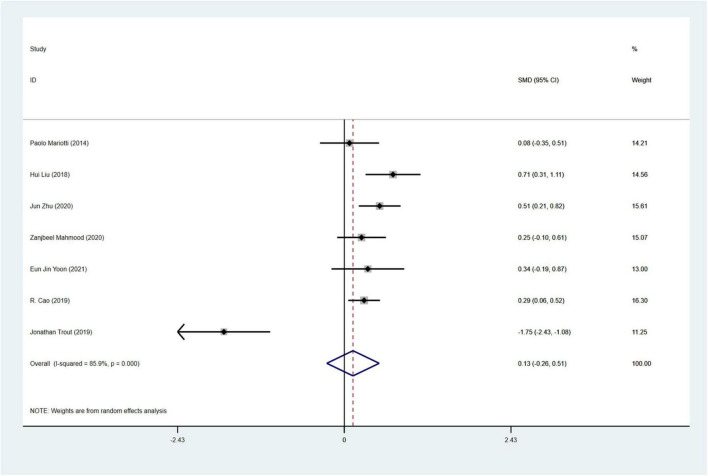
Forest plot based on the difference in the anxious severity among Parkinson’s disease patients with or without rapid eye movement sleep behavior disorder (RBD). SMD, Standardized Mean Difference; CI, confidence intervals.

A total of 7 studies18, 29, 48, 57, 58, 61, 66 involving 989 PD patients (RBD group: 468 patients, NRBD group: 521 patients) were included in the meta-analysis of anxiety. In this study, Hamilton Anxiety Scale (Ham-A) and the State-Trait Anxiety Inventory (STAI) were adopted to assess anxiety. Due to the variety of tools used to assess anxiety, subgroup analyses were conducted based on the assessment tools. The heterogeneity among the studies was high (I^2^ = 85.9%), therefore, a random-effects model was employed for integration. The results showed that PD patients in the RBD group had more severe anxiety symptoms than those in the NRBD group (SMD = 0.13, 95% CI: [−0.26, 0.51], *P* < 0.001) (Figure 6). Further subgroup analyses (Table 2) based on RBD type, RBD diagnostic tools, geographic regions, and the type of anxiety rating scales all indicated that the study by Jonathan Trout57 was a source of heterogeneity. After excluding this study, the homogeneity of the remaining studies improved (I^2^ = 21%), and a fixed-effects model was used for re-analysis, showing that the RBD group had more severe anxiety symptoms compared to the NRBD group (SMD = 0.36, 95% CI: [0.20, 0.52], *P* = 0.276).

**TABLE 2:** Subgroup analysis based on anxiety in PD patients with or without RBD.

Subgroup	Number of studies	SMD	95% CI	*P*	I2
**Anxiety**					
**RBD diagnose**					
PSG	2	0.32	(−0.10, 0.75)	0.139	62.4%
RBDSQ	5	0.03	(−0.53, 0.58)	0.924	89.8%
**geographic location**					
Asia	3	0.46	(0.23, 0.70)	0.000	44.6%
Americas	3	0.13	(−0.26, 0.51)	0.533	93.2%
**RBD types**					
RBD	2	0.32	(−0.10, 0.75)	0.139	62.4%
pRBD	5	0.13	(−0.26, 0.51)	0.924	89.8%
**Test types**					
Ham-A	3	0.45	(0.12, 0.78)	0.008	57.3%
STAI	4	0.13	(−0.26, 0.51)	0.642	90.9%

#### 3.4.4 Analysis of depression

**FIGURE 7:**
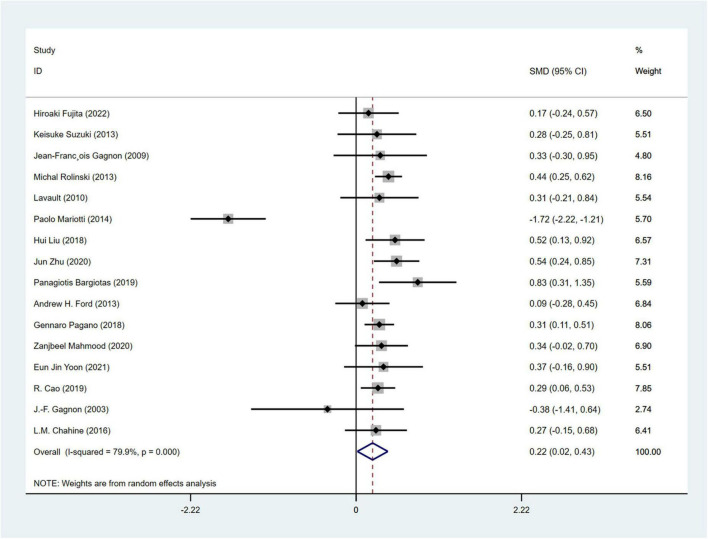
Forest plot based on difference in the depression severity among Parkinson’s disease patients with or without rapid eye movement sleep behavior disorder (RBD). SMD, Standardized Mean Difference; CI, confidence intervals.

A total of 16 studies (Gagnon et al., 2004, 2009; Lavault et al., 2010; Ford et al., 2013; Suzuki et al., 2013; Rolinski et al., 2014; Mariotti et al., 2015; Chahine et al., 2016; Pagano et al., 2018; Bargiotas et al., 2019; Liu et al., 2019; Cao et al., 2020; Mahmood et al., 2020; Yoon and Monchi, 2021; Zhu et al., 2021; Fujita et al., 2022) involving 2,511 PD patients (RBD group: 1,030; NRBD group: 1,481) were included in the meta-analysis of depression. In this study, Beck Depression Inventory (BDI), Beck Depression Inventory-II (BDI-II), Hamilton Depression Scale (HAMD), and Geriatric Depression Scale (GDS) were used to assess depression. Due to the variety of tools used to assess depression, subgroup analyses were conducted based on the assessment tools. The heterogeneity among the studies was relatively high (I^2^ = 79.9%), therefore, a random-effects model was employed for integration. The results indicated that the RBD group had higher depression scores compared to the NRBD group (SMD = 0.22, 95% CI: [0.02, 0.43], *P* < 0.001) (Figure 7). The funnel plot (Supplementary Figure 2) and Begg’s test (*P* = 0.260) suggested no publication bias; sensitivity analysis did not identify a source of heterogeneity. Subgroup analyses (Table 3) indicated that the RBD type, the diagnostic tools for RBD, the type of depression rating scales, and the geographic origin of the study population might be sources of heterogeneity in the analysis of depression symptoms.

**TABLE 3:** Subgroup analysis based on depression in PD patients with or without RBD.

Subgroup	Number of studies	SMD	95% CI	*P*	I2
**Depression**					
**RBD diagnose**					
PSG	4	−0.17	(−1.37, 1.03)	0.780	95.5%
RBDSQ	10	0.35	(0.25, 0.44)	<0.001	0.0%
**geographic location**					
Asia	5	0.37	(0.22, 0.52)	<0.001	0.0%
Europe	5	0.05	(−0.64, 0.74)	0.889	0.889
Americas	5	0.29	(0.13, 0.46)	<0.001	0.0%
**RBD types**					
RBD	5	−0.07	(−1.03, 0.89)	0.887	94.0%
pRBD	11	0.33	(0.24, 0.42)	<0.001	0.0%
**Test types**					
BDI	2	0.42	(0.25, 0.60)	<0.001	0.0%
BDI-II	3	0.23	(−0.05, 0.52)	0.110	0.0%
HAMD	4	0.05	(−0.95, 1.05)	0.919	95.6%
GDS	7	0.28	(0.16, 0.40)	<0.001	0.0%

#### 3.4.5 Analysis of sleep disorder

**FIGURE 8:**
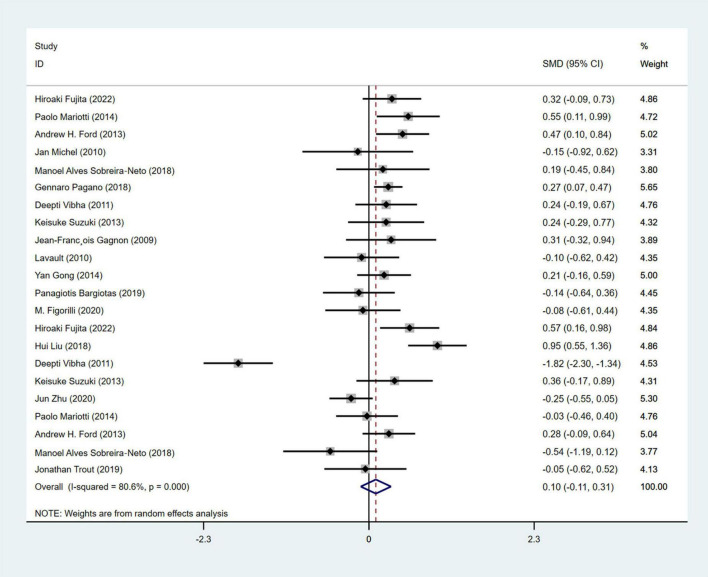
Forest plot based on difference in the sleep quality among Parkinson’s disease patients with or without rapid eye movement sleep behavior disorder (RBD). SMD, Standardized Mean Difference; CI, confidence intervals.

A total of 16 studies(1, 17, 20, 23, 25, 33, 35, 42, 45, 47, 54, 56, 64, 65, 67, 70) (Gagnon et al., 2009; Benninger et al., 2010; Iranzo et al., 2011; Ford et al., 2013; Suzuki et al., 2013; Ferri et al., 2014; Gong et al., 2014; Mariotti et al., 2015; Pagano et al., 2018; Sobreira-Neto et al., 2018; Bargiotas et al., 2019; Cabreira and Massano, 2019; Liu et al., 2019; Trout et al., 2019; Zhu et al., 2021; Fujita et al., 2022) with 1,509 PD patients (RBD group: 709, NRBD group: 800) were included in the meta-analysis concerning sleep conditions. The Epworth Sleepiness Score (ESS), Parkinson’s Disease Sleep Scale (PDSS), Parkinson’s Disease Sleep Scale-2 (PDSS-2), and Pittsburgh Sleep Quality Index (PSQI) were used to assess the sleep quality of PD patients in the included studies. Among them, 6 studies (Vibha et al., 2011; Ford et al., 2013; Suzuki et al., 2013; Mariotti et al., 2015; Sobreira-Neto et al., 2018; Fujita et al., 2022) utilized two different sleep assessment scales for evaluating the sleep quality of PD patients. Due to the diversity of tools used to assess sleep disorder, subgroup analyses were conducted based on the assessment tools. The analysis showed relatively high heterogeneity among the studies (I^2^ = 80.6%), and a random-effects model was employed for integration. The results indicated that patients in the RBD group had higher scores for sleep disorder compared to those in the NRBD group (SMD = 0.10, 95% CI: [−0.11, 0.31], *P* < 0.001) (Figure 8), and the funnel plot and Begg’s test (*P* = 0.114) suggested no publication bias among the studies. Sensitivity analysis indicated good stability across the studies. Subgroup analyses (Table 4) suggested that the geographic origin of the study population, the type of sleep disorder rating scales, the RBD type, and the diagnostic tools might be sources of heterogeneity in the analysis of sleep disorder.

**TABLE 4:** Subgroup analysis based on sleep disorder in PD patients with or without RBD.

Subgroup	Number of studies	SMD	95% CI	*P*	I2
**Sleep disorder**					
**RBD diagnose**					
ICSD	3	−0.43	(−1.83, 0.98)	0.552	95.7%
PSG	9	−0.01	(−0.21, 0.20)	0.962	39.8%
RBDSQ	8	0.35	(0.13, 0.56)	0.002	53.5%
Other	2	0.37	(0.11, 0.63)	0.005	0.0%
**geographic location**					
Asia	6	0.09	(−0.37, 0.56)	0.692	91.2%
Europe	5	0.04	(−0.19, 0.28)	0.726	22.5%
Americas	4	0.11	(−0.16, 0.38)	0.433	35.2%
Oceania	1	0.37	(0.11, 0.63)	0.005	0.0%
**RBD types**					
RBD	9	−0.12	(−0.47, 0.22)	0.481	83.7%
pRBD	7	0.35	(0.19, 0.52)	<0.001	42.3%
**Test types**					
ESS	13	0.24	(0.12, 0.35)	<0.001	0.0%
PDSS	3	−0.57	(−1.70, 0.56)	0.325	41.4%
PDSS-2	2	0.78	(0.38, 1.14)	<0.001	95.2%
PSQI	4	−0.01	(−0.32, 0.30)	0.945	37.4%

## 4 Discussion

To the best of our knowledge, this study is the first systematic meta-analysis comparing the differences in motor and non-motor symptoms between PD patients with and without RBD. Through the extensive database search, a total of 53 articles encompassing 3,084 PD patients with RBD were analyzed. The results revealed that PD patients in the RBD group experienced more severe motor symptoms, including higher UPDRS-III scores and Hoehn and Yahr stages, compared to the NRBD group. Additionally, RBD patients exhibited a greater range of non-motor symptoms, such as cognitive impairment, psychological health impacts, and increased incidence of sleep disorder and visual hallucinations. Sensitivity analysis confirmed the stability and reliability of these findings.

The meta-analysis found that PD patients with RBD have higher Hoehn and Yahr stages and UPDRS-III scores, indicating a more severe disease condition and faster progression compared to those without RBD. These findings are consistent with previous reports (Zhu et al., 2021). As PD progresses, the dopaminergic neurotransmission in the striatum weakens, which leads to a reduction in dopamine regulatory function, resulting in motor control impairments. PD patients with RBD often exhibit more severe and widespread neurodegeneration in the substantia nigra of the midbrain (Iranzo et al., 2011), further diminishing dopaminergic neurons and exacerbating motor symptoms. The relatively high heterogeneity in studies on PD-RBD motor symptoms could be related to the diagnosis criteria for RBD. Many patients diagnosed with cRBD were not tested with PSG (Stefani and Högl, 2020) but diagnosed through the 2005 ICSD criteria (Thorpy, 2012), potentially introducing variability due to the questionnaire’s design limitations and evaluator subjectivity, suggesting the need for more standardized diagnosis procedures in future research.

Moreover, the study found that cognitive functions in PD patients were affected by RBD, with lower MMSE scores observed in the RBD group, indicating more severe cognitive impairments, which aligns with findings from Xie et al. (2021). The mechanism behind this phenomenon may be related to the shared pathological basis of cognitive impairment in patients with both RBD and PD. The structures involved in controlling the onset of REM sleep are located in the periaqueductal gray area near the aqueduct of the midbrain and in the anterior part of the locus coeruleus, the sublaterodorsal nucleus, and dorsal raphe nucleus (Jiménez-Jiménez et al., 2021). A study by Meyer et al. (2009) found that the worsening of cognitive impairment in PD patients is associated with a reduction in acetylcholine receptors in various brain regions, including the thalamus, midbrain, pons, hippocampus, and cerebellum. Given that this study solely utilized the MMSE for cognitive assessment, it might be challenging to detect mild cognitive impairments or impairments due to other causes during the evaluation process. For future research, it’s recommended that researchers combine patient MR imaging and clinical manifestations to interpret more precise cognitive assessment results.

According to research (Meral et al., 2007), visual hallucination is a common symptom of PD, with PD patients with RBD showing a higher likelihood of experiencing visual hallucination compared to those without RBD. This finding aligns with the results of this study. Although the mechanisms behind the occurrence of visual hallucinations may be associated with the ascending cholinergic system and the serotonin release involved in the sleep-wake cycle, and abnormal discharges in the brain cortex areas processing visual information (Gelb et al., 1999), the use of certain medications such as dopamine receptor agonists and anticholinergic drugs (Sanchez-Ramos et al., 1996) may also be related to the occurrence of visual hallucination in PD patients. Therefore, the mechanism behind the occurrence of visual hallucination in PD patients with RBD remains unclear, and more basic experiments are required in future research to confirm its mechanism.

Previous research has reported an association between PD and the onset of psychiatric disorders, including anxiety and depression disorders. One mechanism is that damage to the limbic system can lead to a reduction in neurotransmitters such as dopamine and norepinephrine, which may exacerbate psychiatric symptoms. Another mechanism is that the accumulation of Lewy bodies in the midbrain raphe nuclei can lead to a decrease in the serotonin level in the body (Iranzo et al., 2011). In our study, it was observed that patients in the RBD group exhibited more severe symptoms of anxiety and depression compared to those in the NRBD group, which is consistent with previous reports (Mahmood et al., 2020). The underlying reason may be related to the anatomical overlap between the occurrence of anxiety, depression, and RBD. The onset of RBD is associated with structures such as the raphe nucleus and locus coeruleus, which are anatomically linked to the pathogenesis of anxiety and depression. In the analysis of anxiety symptoms, it was found that the study by Jonathan Trout (Trout et al., 2019) was a source of heterogeneity. After excluding this study, the homogeneity among the remaining studies improved, and the PD-RBD group exhibited markedly more severe anxiety symptoms than the NRBD group. For depression symptoms, factors such as RBD type, RBD diagnostic tools, geographic regions, and the type of depression rating scales were considered potential sources of heterogeneity among studies. Future research should employ larger sample sizes and more precise scoring criteria to assess the occurrence of psychiatric disorders in PD patients.

In this study, we also discovered that the RBD group had more sleep issues compared to the NRDB group, consistent with previous reports. This may be attributed to multiple factors. On one hand, PD patients with RBD often experience excessive loss of dopamine neurons, leading to an imbalance in neurotransmitter projections of the ascending reticular activating system, causing disturbances in the ascending arousal system. On the other hand, PD patients with RBD often display abnormal sleep behaviors at night, such as kicking, excessive clenching of the jaw, and twitching, which often disrupt sleep and lead to daytime somnolence and nighttime insomnia, inducing rhythm sleep disorders. Moreover, the patients often have comorbid psychiatric disorders, which may further exacerbate their sleep issues (Raggi et al., 2013). In this study, the sources of heterogeneity in the analysis of sleep disorders in RBD may mainly stem from the geographic origin of the study population, the type of sleep disorder rating scales, the RBD type, and the diagnostic tools. Future research should focus on larger-scale clinical studies to elucidate the relationship between RBD and sleep disorders in patients with PD. In summary, RBD may correlate with degeneration in the locus coeruleus, subcoeruleus area, and adjacent areas at the midbrain-pontine junction, as well as the midbrain-striatal dopaminergic neuronal pathways. Anatomically, these structures are interconnected with PD, potentially exacerbating both motor and non-motor symptoms in PD patients with RBD. Clinically, it is crucial to monitor PD patients with concurrent RBD and intervene with prompt treatments, thereby enhancing the overall quality of life for these patients.

Some limitations exist in this study. Firstly, the diagnostic tools for cRBD in patients included in the PD-RBD group are diverse, and most studies did not implement PSG monitoring. Such a diagnostic approach may affect the reliability of the results of PD-related symptoms. Secondly, due to the small sample size in some studies and limitations in study design, it was not possible to include enough data samples for further analysis of more motor symptoms (such as bradykinesia, rigidity, and gait impairment) and some non-motor symptoms (like constipation, sweating, and orthostatic hypotension) in PD patients Additionally, the evaluation of mental health (anxiety and depression) and sleep disorder used various measurement scales, which could contribute to relatively high heterogeneity among studies. Ultimately, the evaluation of motor and non-motor symptoms necessitates the adoption of more uniformly standardized tools.

## 5 Conclusion

In conclusion, our study results indicated that PD patients with RBD can exhibit more severe motor and non-motor symptoms. Clinicians should identify the presence of RBD in PD patients early and take proactive interventions for better treatment outcomes. In future research, larger sample sizes and more uniform RBD diagnostic criteria should be adopted to convincingly demonstrate the correlation of RBD to the progression of PD and the potential pathological mechanisms involved.

## Data availability statement

The original contributions presented in this study are included in the article/Supplementary material, further inquiries can be directed to the corresponding author.

## Author contributions

WZ: Conceptualization, Writing – original draft, Writing – review and editing. YP: Methodology, Writing – original draft. KL: Formal analysis, Investigation, Writing – original draft. KT: Formal analysis, Investigation, Writing – original draft. QW: Resources, Writing – original draft. YY: Supervision, Writing – original draft.

## Supplementary material

Supplementary Figure 1 Funnel plot displaying the probable publication bias for estimated UPDRS-III **(A)** and Hoehn and Yahr stage **(B)** between PD patients with and without RBD.

Supplementary Figure 2 Funnel plot displaying the probable publication bias for estimated MMSE **(A)**, hallucination **(B)**, anxiety, **(C)**, depression, and **(D)**, sleep disorder **(E)** between PD patients with and without RBD.
