# Supplementary material for: The correlation between rapid eye movement sleep behavior disorder and the progress of Parkinson’s disease: a systematic review and meta-analysis
Source: Front Aging Neurosci. 2024 Jul 17;16:1418751. doi: 10.3389/fnagi.2024.1418751 (PMC11288858; doi:10.3389/fnagi.2024.1418751)
Supplement: Supplementary file 1 [file Table_1.DOCX]

**Table S1.** Literature search strategy

1.Pubmed

| Search number | Query | Records |
| --- | --- | --- |
| #1 | REM Sleep Behavior Disorder[MeSH Terms] | 1839 |
| #2 | "rapid eye movement behavi*"[Title/Abstract] OR " rapid eye movement sleep behavi*"[Title/Abstract] OR " rapid eye movement sleep behavior disorder"[Title/Abstract] OR " REM behavi*"[Title/Abstract] OR " REM sleep behavi*"[Title/Abstract] OR " REM Sleep Behavior Disorder"[Title/Abstract] | 3056 |
| #3 | ("rapid eye movement behavi*"[Title/Abstract] OR " rapid eye movement sleep behavi*"[Title/Abstract] OR " rapid eye movement sleep behavior disorder"[Title/Abstract] OR " REM behavi*"[Title/Abstract] OR " REM sleep behavi*"[Title/Abstract] OR " REM Sleep Behavior Disorder"[Title/Abstract]) OR (REM Sleep Behavior Disorder[MeSH Terms]) | 3274 |
| #4 | Parkinson disease[MeSH Terms] | 82196 |
| #5 | "Parkinson’s disease"[Title/Abstract] OR " Primary Parkinsonism"[Title/Abstract] OR " Paralysis Agitans"[Title/Abstract] OR " idiopathic parkinsonism"[Title/Abstract] OR " Lewy bodies of Parkinsons disease"[Title/Abstract] OR " Lewy body Parkinsons disease"[Title/Abstract] | 108498 |
| #6 | ("Parkinson’s disease"[Title/Abstract] OR " Primary Parkinsonism"[Title/Abstract] OR " Paralysis Agitans"[Title/Abstract] OR " idiopathic parkinsonism"[Title/Abstract] OR " Lewy bodies of Parkinsons disease"[Title/Abstract] OR " Lewy body Parkinsons disease"[Title/Abstract]) OR (Parkinson disease[MeSH Terms]) | 81070 |
| #7 | #3 AND #6 | 1761 |

2.Cochrane

| Search number | Query |
| --- | --- |
| #1 | MeSH descriptor: [REM Sleep Behavior Disorder] explode all trees |
| #2 | (rapid eye movement sleep behavior disorder):ti,ab,kw OR (REM Sleep Behavior Disorder):ti,ab,kw OR (Behavior Disorder, REM):ti,ab,kw OR (Behavior Disorders, REM):ti,ab,kw OR (Behavior Disorders, REM):ti,ab,kw |
| #3 | (Rapid Eye Movement Sleep Behavior Disorder):ti,ab,kw OR (rapid eye movement behaviour disorder):ti,ab,kw |
| #4 | #1 or #2 or #3 |
| #5 | MeSH descriptor: [Parkinson Disease] explode all trees |
| #6 | ("Parkinson's disease"):ti,ab,kw OR ("Parkinson's disease dementia"):ti,ab,kw OR (Idiopathic Parkinson Disease):ti,ab,kw |
| #7 | #5 or #6 |
| #8 | #4 and #7 |

1. **Embase**

| Search number | Query | Records |
| --- | --- | --- |
| #1 | 'rem sleep behavior disorder'/exp | 349 |
| #2 | 'rapid eye movement'/exp OR 'rapid eye movement' OR (('rapid'/exp OR rapid) AND ('eye'/exp OR eye) AND ('movement'/exp OR movement) AND behavi*) OR 'rapid eye movement sleep behavi*':ab,ti OR 'rapid eye movement sleep behavior disorder':ab,ti OR 'rem sleep behavi*':ab,ti OR 'rem sleep behavior disorder':ab,ti | 39807 |
| #3 | #1 OR #2 | 39869 |
| #4 | 'parkinson disease'/exp | 196116 |
| #5 | 'parkinson s disease' OR (parkinson AND ('s'/exp OR s) AND ('disease'/exp OR disease)) OR 'primary parkinsonism':ab,ti OR 'paralysis agitans':ab,ti OR 'idiopathic parkinsonism':ab,ti OR 'lewy bodies of parkinsons disease':ab,ti OR 'lewy body parkinsons disease':ab,ti | 216374 |
| #6 | #4 OR #5 | 241974 |
| #7 | #3 AND #6 | 2636 |

**4.Web of science**

| Search number | Query |
| --- | --- |
| #1 | TS=(REM sleep behavior disorder ) OR TS=(rapid eye movement behavi* ) OR TS=(rapid eye movement sleep behavi*) OR TS=(rapid eye movement sleep behavior disorder) OR TS=(REM behavi*) OR TS=(REM sleep behavi*) OR TS=(REM Sleep Behavior Disorder) |
| #2 | TS=(Parkinson disease) OR TS=(Parkinson’s disease) OR TS=(Primary Parkinsonism) OR TS=(Paralysis Agitans) OR TS=(idiopathic parkinsonism) OR TS=(Lewy bodies of Parkinsons disease) OR TS=(Lewy body Parkinsons disease) |
| #3 | #1 AND #2 |
