# Supplementary material for: The correlation between rapid eye movement sleep behavior disorder and the progress of Parkinson’s disease: a systematic review and meta-analysis
Source: Front Aging Neurosci. 2024 Jul 17;16:1418751. doi: 10.3389/fnagi.2024.1418751 (PMC11288858; doi:10.3389/fnagi.2024.1418751)
Supplement: Supplementary file 2 [file Table_2.DOCX]

**Table S2** Subgroup analysis based on motor symptoms (UPDRS-III, Hoehn and Yahr stage) between PD patients with or without RBD

| Subgroup | Number of studies | SMD | 95% CI | p | I^2^ |
| --- | --- | --- | --- | --- | --- |
| **UPDRS-III** | | | | | |
| **RBD diagnose** |  |  |  |  |  |
| ICSD | 9 | 0.24 | (0.08, 0.41) | 0.004 | 0.0% |
| PSG | 13 | 0.24 | (-0.02, 0.49) | 0.067 | 67.8% |
| RBDSQ | 14 | 0.15 | (0.03,0.28) | 0.013 | 61.4% |
| Other | 4 | 0.26 | （-0.08,0.59） | 0.139 | 55.4% |
| **geographic location** |  |  |  |  |  |
| Asia | 15 | 0.27 | （0.13,0.40） | <0.001 | 55.4% |
| Europe | 12 | 0.22 | （0.05,0.38） | 0.01 | 35.7% |
| Americas | 13 | 0.13 | （-0.06,0.31） | 0.178 | 56.1% |
| **RBD kinds** |  |  |  |  |  |
| cRBD | 23 | 0.24 | （0.09,0.39） | 0.001 | 47.7% |
| pRBD | 18 | 0.20 | （0.11,0.29） | 0.003 | 58.7% |
| **H-Y** | | | | | |
| **RBD diagnose** |  |  |  |  |  |
| ICSD | 4 | 0.05 | （-0.31,0.42） | 0.769 | 79.3% |
| PSG | 11 | 0.37 | （-0.33,1.07） | 0.298 | 93.4% |
| RBDSQ | 6 | 0.15 | （-0.04,0.35） | 0.128 | 54.4% |
| **geographic location** |  |  |  |  |  |
| Asia | 11 | 0.60 | （0.15，1.04） | 0.008 | 56.7% |
| Europe | 6 | -0.02 | （-0.31,0.27） | 0.901 | 94.3% |
| Americas | 6 | -0.01 | （-0.26,0.24） | 0.936 | 0.0% |
| **RBD kinds** |  |  |  |  |  |
| cRBD | 14 | 0.36 | （-0.15,0.86） | 0.165 | 91.8% |
| pRBD | 9 | 0.29 | （0.03,0.55） | 0.151 | 76.1% |

**Table S3** Subgroup analysis for non-motor symptoms (MMSE) between PD patients with or without RBD

| Subgroup | Number of studies | SMD | 95% CI | p | I^2^ |
| --- | --- | --- | --- | --- | --- |
| **MMSE** | | | | | |
| **RBD diagnose** |  |  |  |  |  |
| ICSD | 4 | -0.21 | (-0.40,-0.03) | 0.025 | 52.6% |
| PSG | 13 | -0.44 | (-0.78,-0.09) | 0.014 | 81.4% |
| RBDSQ | 6 | 0.02 | (-0.43,0.47) | 0.938 | 91.0% |
| Other | 3 | -0.55 | (-1.56,0.46) | 0.285 | 93.4% |
| **geographic location** |  |  |  |  |  |
| Asia | 14 | -0.23 | (-0.48,0.01) | 0.059 | 87.1% |
| Europe | 8 | -0.21 | (-0.43,0.01) | 0.063 | 47.4% |
| Americas | 5 | -0.92 | (-1.93,0.10) | 0.076 | 89.0% |
| **RBD kinds** |  |  |  |  |  |
| RBD | 17 | -0.38 | (-0.62,-0.15) | 0.001 | 78.2% |
| pRBD | 11 | -0.30 | (-0.48,-0.11) | 0.246 | 89.5% |
