# Supplementary figures and images for: The correlation between rapid eye movement sleep behavior disorder and the progress of Parkinson’s disease: a systematic review and meta-analysis
Source: Front Aging Neurosci. 2024 Jul 17;16:1418751. doi: 10.3389/fnagi.2024.1418751 (PMC11288858; doi:10.3389/fnagi.2024.1418751)

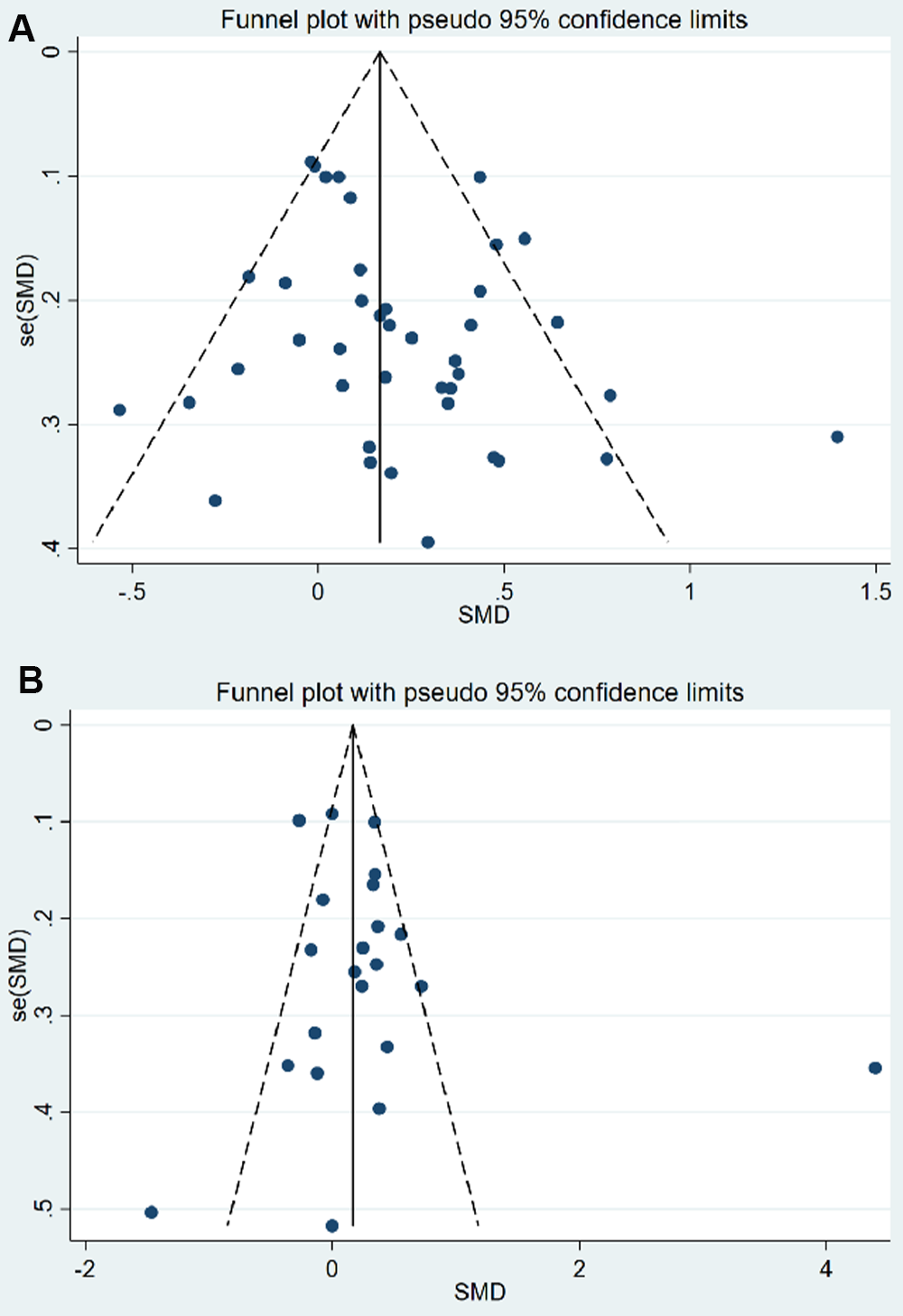

Supplement: Supplementary Figure 1 — Funnel plot displaying the probable publication bias for estimated UPDRS-III (A) and Hoehn and Yahr stage (B) between PD patients with and without RBD. [file Image_1.TIF]

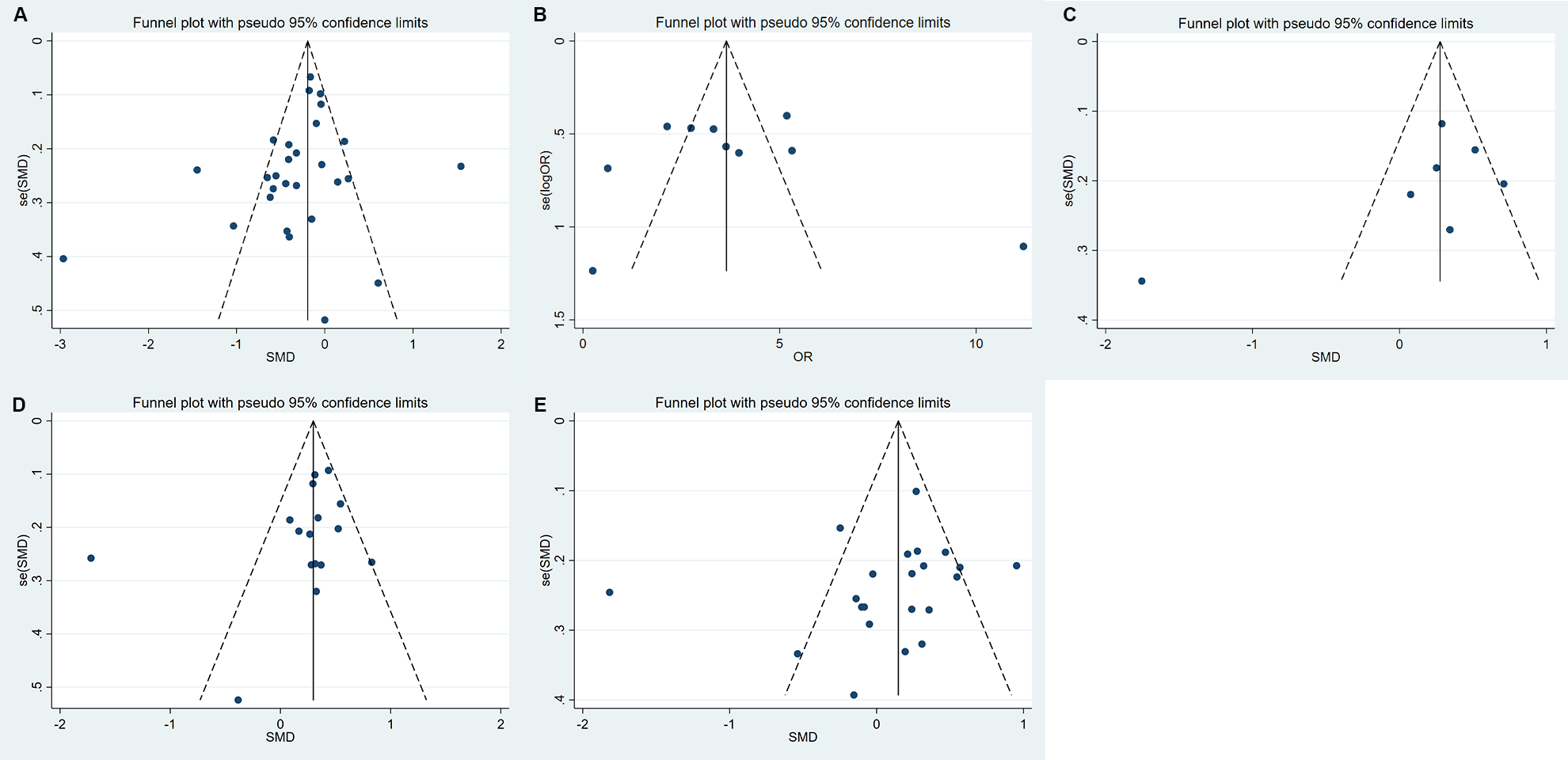

Supplement: Supplementary Figure 2 — Funnel plot displaying the probable publication bias for estimated MMSE (A), hallucination (B), anxiety, (C), depression, and (D), sleep disorder (E) between PD patients with and without RBD. [file Image_2.TIF]
